# Extracellular diffusion quantified by magnetic resonance imaging during rat C6 glioma cell progression

**DOI:** 10.1590/1414-431X20175403

**Published:** 2017-07-03

**Authors:** G. Song, T. Luo, L. Dong, Q. Liu

**Affiliations:** 1 Department of Radiology, The First Affiliated Hospital, Chongqing Medical University, Chongqing, China; 2 Department of Radiology, The Secondary Affiliated Hospital, Baotou Medical College, Baotou, Inner Mongolia, China

**Keywords:** C6 glioma model, Extracellular space, Magnetic resonance imaging, Rats

## Abstract

Solution reflux and edema hamper the convection-enhanced delivery of the standard treatment for glioma. Therefore, a real-time magnetic resonance imaging (MRI) method was developed to monitor the dosing process, but a quantitative analysis of local diffusion and clearance parameters has not been assessed. The objective of this study was to compare diffusion into the extracellular space (ECS) at different stages of rat C6 gliomas, and analyze the effects of the extracellular matrix (ECM) on the diffusion process. At 10 and 20 days, after successful glioma modeling, gadolinium-diethylenetriamine pentaacetic acid (Gd-DTPA) was introduced into the ECS of rat C6 gliomas. Diffusion parameters and half-life of the reagent were then detected using MRI, and quantified according to the mathematical model of diffusion. The main ECM components [chondroitin sulfate proteoglycans (CSPGs), collagen IV, and tenascin C] were detected by immunohistochemical and immunoblot analyses. In 20-day gliomas, Gd-DTPA diffused more slowly and derived higher tortuosity, with lower clearance rate and longer half-life compared to 10-day gliomas. The increased glioma ECM was associated with different diffusion and clearance parameters in 20-day rat gliomas compared to 10-day gliomas. ECS parameters were altered with C6 glioma progression from increased ECM content. Our study might help better understand the glioma microenvironment and provide benefits for interstitial drug delivery to treat brain gliomas.

## Introduction

Glioblastoma is the most common type of primary brain tumor, accounting for 81% of all malignant brain tumors (1) and often leading to significant morbidity and mortality. Within the past few decades, the treatment method for glioma has improved, and radiation combined with chemotherapy is used as adjuvant therapy after surgery (2). However, most patients with glioblastoma have only 7–10 months of progression-free survival (3). Glioblastoma has a persistent characteristic and always has a poor prognosis. As most chemotherapeutic drugs cannot pass the blood-brain barrier, drugs used to reach the tumor are ineffective (4). Several new treatment strategies have been proposed, including local medication treatments that can directly bypass the blood-brain barrier, such as local injection, convection-enhanced drug delivery (CED), and several types of embedded polymers. Local treatment allows drugs with high bioavailability to directly target the tumor with little medication loss (5); however, solution reflux, edema, and other side effects have prevented CED from being developed into a standard treatment for glioma (6).

To overcome this, a method using real-time magnetic resonance imaging (MRI) was developed to monitor the treatment dosing process (7,8); however, local diffusion and clearance parameters have not been quantitatively assessed. In a previous study, we applied nuclear magnetic resonance (NMR) tracer technology to quantitatively measure the normal rat caudate nucleus, thalamus, occipital cortex proliferation, substantia nigra, and clearance parameters (9,10). The current study firstly applied NMR tracer technology to quantitatively compare diffusion and clearance parameters in 10- and 20-day C6 glioma models, analyzing factors that affected diffusion and clearance of extracellular matrix (ECM) components by immunohistochemistry and western blot.

## Material and Methods

### Cell culture

C6 cells were obtained from the Cell Culture Collection of Chinese Academy of Sciences (Shanghai, China), and cultured and stored according to the provider’s instructions.

### Solution preparation and experimental animals

This study was approved by the Animal Ethics Committee of Baotou Medical College, China. Serial 2× dilution of gadolinium-diethylenetriamine pentaacetic acid (Gd-DTPA; Magnevist, Bayer Healthcare Pharmaceuticals, Germany) were performed to obtain 10 mM. Sixteen adult male Sprague Dawley rats, clean grade, body weight 250–300 g (Baotou Medical College Department of Laboratory Animal Science, license SCXK, Beijing, China, 2011–0012; experimental animals use license SYXK (character), 2011–0039) were randomly divided into 10-day (n=8) and 20-day (n=8) glioma groups. Rats were anesthetized by peritoneal injection of sodium pentobarbital, ethanol, chloral hydrate, magnesium sulfate, and propylene glycol mixture (3.0 mL/kg) and immobilized on a stereotaxic instrument (Stoelting Co., USA). An incision was made along the sagittal suture in the scalp from the area between the ears to the midline area between the eyes, and the meningeal chimney was carefully separated before exposure. Drilling was positioned on the right side of the skull surface (1.0 mm from the chimney; 3.5 mm open right side). The C6 cell suspension (5×10^6^ cells, in 10 μL) was slowly injected into the skull (10 min) on the right side of the caudate nucleus using a Trace injector needle. Skull holes were then sealed with bone wax, and the scalp was sutured.

### Tracer diffusion model of rat C6 glioma cell clearance

Rats were anesthetized as mentioned above, and anesthesia with 10% chloral hydrate (3.5 mL/kg) was maintained throughout the experimental period (about 0.7 mL·kg^-1^·h^-1^). The animals were maintained on a heating pad at 38.0±0.5°C. Rats were pre-scanned on an MRI (Siemens, Germany) system using wrist coils, with T1-weighted three-dimensional (3D) magnetization for quick-acquisition gradient echo sequence (Tl 3 d MP-RAGE) scanning (11). After MRI was processed and the puncture site determined, the skin and cranial area were disinfected with iodine to prepare for surgery, which was conducted as above. Drill holes were repositioned on the skull surface according to the tumor growth status displayed by MRI pre-scan. Gd-DTPA (10 mM; 2.0 μL) was injected by the same staff member into the center of the tumor area (injection time10 min). Then, MRI was performed at different time points after injection of Gd-DTPA (15 min, 30 min, and 6 h). Imaging sequences and parameters were the same as those mentioned above.

### Image post-processing and parameter calculations

Subtraction images were obtained by matching pre-injection and post-injection scanning images using MATLAB7.8 software (MathWorks, USA) (12). Our preliminary study using 3.0 T T1-weighted NMR and 3D MP-RAGE scanning showed a linear relationship between signal strength (ΔSI) and Gd-DTPA concentration (11). Previous 1/T1 values compared with Gd-DTPA concentrations showed that such relationship was more conducive to real-time imaging computing (13–15). The quantitative extracellular interstitial diffusion MRI tracer method was used for the main parameters, including the free diffusion coefficient (D), effective diffusion coefficient (D*), and infusion and clearance rate constant (K). Li et al. (16) first reported the parameter calculation method in 2013. For measuring brain tissue, the fluid-flow half-life should be higher than two standard deviations of background noise of the signal strength used as the threshold. The region of interest can be extracted step by step using all the pixels to obtain total Gd-DTPA scan time (Gd_sum_). According to first-order kinetics constants, the half-life of Gd-DTPA can be obtained by curve fitting (Gd_sum_=e^-Kt^), and the half-life can be derived as (2)/K.

### Immunohistochemical assay

Common extracellular matrix molecules were selected for immunohistochemical analysis (17–19). Five-micrometer paraffin embedded sections were dewaxed, and treated with 3% hydrogen peroxide after antigen retrieval. Then, rabbit anti-rat primary antibodies against chondroitin sulfate proteoglycan, key raw tenascin C, and type IV collagen (Beijing Boosen Biological Technology Co., Ltd., China; 1:200) were added overnight at 4°C. This was followed by incubation with secondary antibody (1:200; Bioss Inc., USA) at room temperature for 30 min. The DAB chromogenic reagent was used for detection, with nuclear counterstaining.

### Western blotting

For western blot (20–22), tumor samples were homogenized, and equal amounts of protein (50 μg) in each sample were separated by 12% sodium dodecyl sulphate-polyacrylamide gel electrophoresis (SDS-PAGE) and transferred onto nitrocellulose membranes. After blocking with 5% skim milk, the membranes were incubated with rabbit anti-rat chondroitin sulfate proteoglycan, key raw tenascin C, type IV collagen (1:1000; Bioss Inc.), and GAPDH (1:10000; Bioss Inc.) primary antibodies at 4°C overnight. After incubation with secondary antibodies at room temperature for 1 h, the membranes were treated with immunoblot chemiluminescence detection reagent (enhanced chemiluminescence), washed, and exposed to X-ray films. ImageJ (National Institutes of Health, USA) was used for analysis. The GAPDH protein was used as an internal control; grey values of bands were determined to compare expression levels as ratios.

### Statistical analysis

SPSS 19 (IBM Corp., USA) was used for statistical analyses. Data are reported as means±SD. Independent samples *t*-test was used to compare two groups for D*, tortuosity (λ), K, and half-life. Paired samples *t*-test was used to compare two groups for protein amounts. P<0.05 was considered to be statistically significant.

## Results

### Gd-DTPA diffusion parameters and half-life in the 10- and 20-day glioma groups

**Figure 1.**
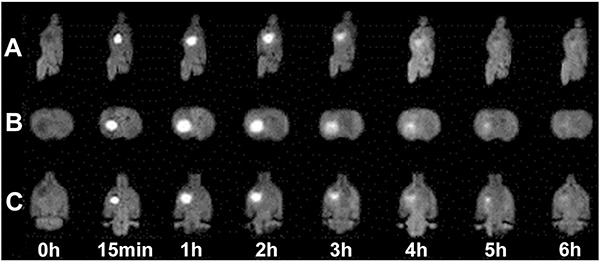
Spatiotemporal distribution of gadolinium-diethylenetriamine pentaacetic acid (Gd-DTPA) injected into the 10-day glioma. Sagittal views (*A*), axial views (*B*), and coronal views (*C*) over time before and after injection of Gd-DTPA.

**Figure 2.**
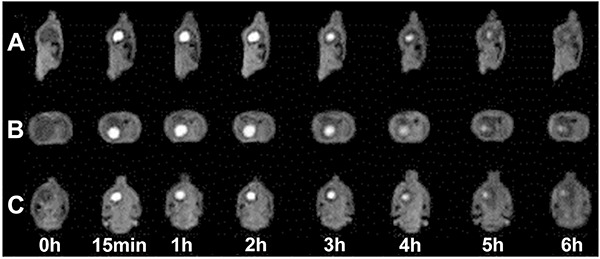
Spatiotemporal distribution of gadolinium-diethylenetriamine pentaacetic acid (Gd-DTPA) injected into the 20-day glioma. Sagittal views (*A*), axial views (*B*), and coronal views (*C*) over time before and after injection of Gd-DTPA.

**Figure 3.**
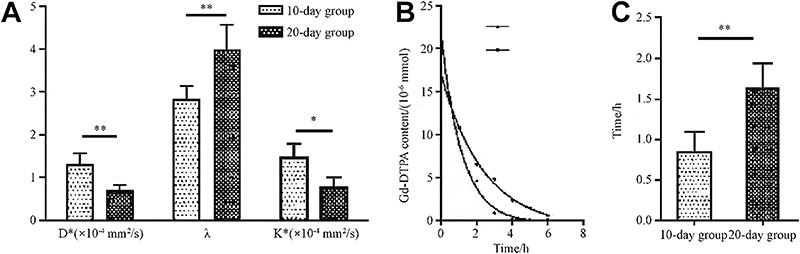
Diffusion parameters and half-life of gadolinium-diethylenetriamine pentaacetic acid (Gd-DTPA) in 10-day (n=8) and 20-day (n=8) gliomas. *A*, Quantitative analyses of diffusion parameters (D*, λ, K). *B*, Amounts of remnant Gd-DTPA in gliomas following an exponential decay. *C*, Statistical analysis of the half-life. Data are reported as means±SD (n=8). *P<0.05; **P<0.01 (*t*-test). D*: effective diffusion coefficient; λ: tortuosity; K: elimination rate constant.

Sagittal, transverse, and coronal MRIs displayed different time points in the Gd-DTPA diffusion process. As shown in Figures 1 and 2, Gd-DTPA was detected outside the cell, with increased local signal strength, before decaying in tracer signal intensity as Gd-DTPA was removed from the extracellular space (at later time points). The 20-day glioma D* value was much smaller than that of 10-day gliomas (6.67±1.78×10^-5^ mm^2^/s *vs* 1.26±0.27×10^-4^ mm^2^/s; t=4.265, P<0.01); this resulted in higher tortuosity (λ) in 20-day gliomas compared with the 10-day glioma group [λ=(D/D*)]^1/2^ (3.99±0.57 *vs* 2.83±0.29; t=4.11, P<0.01; Figure 3A). The K value for the 20-day glioma group (7.67±2.29×10^-5^ mm^2^/s) was less than that of 10-day gliomas (1.46±0.36×10^-4^ mm^2^/s), with a statistically significant difference (t=3.87, P<0.05; Figure 3A). Gd-DTPA half-life in 10-day gliomas (0.86±0.23 h) was significantly reduced compared with that of the 20-day glioma group (1.64±0.12 h; t=5.91; P<0.01; Figure 3C). Finally, apparent diffusion coefficient (ADC) values were extracted from MRI data. Interestingly, 10- and 20-day gliomas showed ADC values of 0.87±0.16×10^-3^ mm^2^/s and 0.52±0.18×10^-3^ mm^2^/s, respectively, indicating a significant decrease between the two time points (P<0.05).

### Expression of extracellular matrix components in 10- and 20-day gliomas

**Figure 4.**
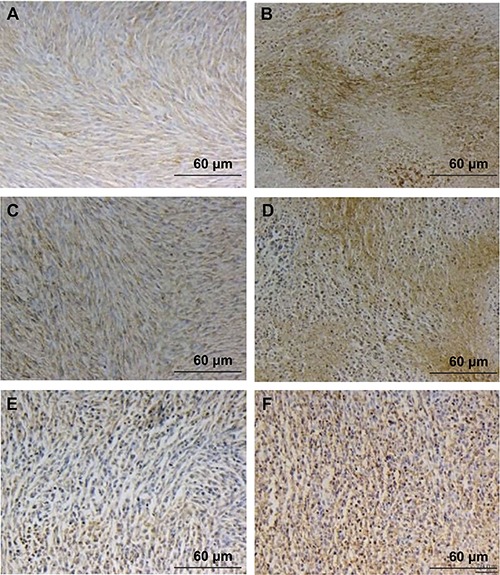
Histological changes of chondroitin sulfate proteoglycans (CSPGs), tenascin C, and collagen IV in the 10- and 20-day gliomas by immunohistochemistry. *A*, CSPGs in a 10-day glioma. *B*, CSPGs in a 20-day glioma. *C*, tenascin C in a 10-day glioma. *D*, tenascin C in a 20-day glioma. *E*, collagen IV in a 10-day glioma. *F*, collagen IV in a 20-day glioma.

**Figure 5.**
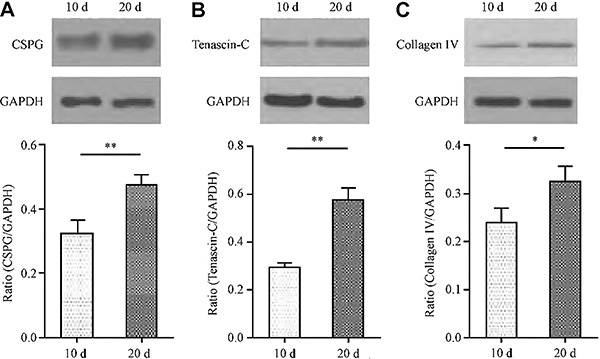
Expression of chondroitin sulfate proteoglycans (CSPGs) in the extracellular matrix in the 10-day and 20-day glioma models by western blotting. *A*, CSPGs in the 10-day (n=6) and 20-day glioma (n=6). *B*, tenascin C in the two-stage models. *C*, collagen IV in the two-stage models. Data are reported as means±SD (n=6). **P<0.01, *P<0.05 (*t*-test).

To better understand the mechanisms behind the altered diffusion parameters (D*, λ, K, and half-life), we compared the levels of glioma chondroitin sulfate proteoglycan, tenascin C, and collagen type IV in 10- and 20-day gliomas by immunohistochemistry (Figure 4). Western blot further confirmed the above findings. Indeed, chondroitin sulfate proteoglycan (CSPGs/GAPDH) expression (Figure 5A) was higher in the 20-day glioma group (0.48±0.07) compared with 10-day gliomas (0.32±0.09; t=4.663; P<0.01); tenascin C/GAPDH was also markedly increased in the 20- compared with the 10-day counterparts (0.58±0.11 *vs* 0.29±0.04; t=6.50; P<0.01; Figure 5B). Finally, type IV collagen/GAPDH in 20-day gliomas (Figure 5C) was higher than in 10-day tumor cells (0.33±0.06 *vs* 0.24±0.07; t=3.81; P<0.05).

## Discussion

It is well known that diffusion weighted MRI (DWI), which follows the diffusion of water molecules, can measure cell organization, brain tumor classification, and tumor judgment, and constitutes the hotspot of current research on radiation and chemotherapy efficacy (23–25). However, because DWI does not distinguish between cells and extracellular diffusion, and has low spatial resolution, it is difficult to obtain a clear picture of diffusion rates using local drugs (26). Real-time magnetic tracers were shown to resolve this issue and have been used successfully.

In both glioma groups, Gd-DTPA signals first increased, followed by a reduction in tracer signal intensity as Gd-DTPA was removed from the extracellular space in the later time points. Interestingly, significant differences were obtained in D*, K, and half-life values between the 10- and 20-day glioma groups. Indeed, lower D* and K, and longer half-life were found in 20-day gliomas. Different properties of glioma cell proliferation and clearance are associated with distinct glioma ECM components; in 20-day gliomas, ECM components such as chondroitin sulfate proteoglycan, tenascin C, and type IV collagen were elevated, in comparison with values obtained for the 10-day glioma group.

Increase in tumor volume is usually an important indicator of tumor progression (27). After tumor cell implantation, tumor volume gradually increases (progression) with time (28). As tumor cells spread they absorb nutrients; therefore, it is crucial that antitumor drugs reach the therapeutic targets in a timely manner (29). ECM components are among the important factors that affect the clearance of tumor cell proliferation in the brain (30,31); they can increase EC space viscosity and reduce the rate of diffusion (32). A viscous ECM increases resistance to molecular motion so that the EC space is no longer a free medium (33). Sim et al. (21) reported that chondroitin sulfate proteoglycan, tendon, tenascin C, and type IV collagen in the glioma cell gap increase as tumor growth progresses (21,34–38). The current findings therefore corroborate other reports. Brain cells outside the gap account for 20% of brain volume, ∼50 nm of the gaps between brain cells within the ECM (34). Researchers now believe that ECM components can reduce the diffusion of solute molecules or decrease membrane molecular diffusion (32). ECM molecules of glioma cells include proteins, polysaccharides, glycoproteins, and collagen (35). This study showed that chondroitin sulfate proteoglycan, tenascin C, and collagen IV were the three main ECS components of a typical glioma cell, and participate in the microenvironment of glioma cell growth to affect its progression and transfer behavior (35). In addition, because several components are often connected between composite structures (35), typical ECM molecules were used to analyze glioma cells outside the gap. The ECS participates in the maintenance of stable conduction of electrical signals among brain cells, formation of transport channels for substances between the cells and blood, and synaptic remodeling (33,36).

This study confirmed that Gd-DTPA diffusion was blocked in rats with C6 glioma. In addition to ECS, this may be associated with tissue architecture as well as size, charge, metabolism, and microvascular permeability of injected substances to the ECM (37). It should be noted that the molecular weight of a substance affects its diffusion, with larger drugs showing lower diffusion rates (38), indicating that other chemotherapeutics used for treating glioma, e.g. tallimustine (697 Da) and carmustine (214 Da), might diffuse faster than Gd-DTPA (938 Da).

### Study limitations

The limitations of this study should be mentioned. The study was very specific to brain cancer, and the present findings should not be generalized to other malignancies. In addition, only typical ECM molecules were selected for analysis. Additional studies are needed to assess other ECM molecules as well as other factors that potentially affect drug diffusion. Finally, a more comprehensive study is warranted for evaluating the effects of diffusion, diffusion parameters of different classes of glioma, and quantitative expression of ECM components.

Brain cancer is one of the main focuses of oncology research. Some scholars believe that tumor size and invasion should be assessed in each phase of glioma treatment. In this study, we found significantly different diffusion parameters in two phases of glioma, suggesting that partial dosing parameters should be adjusted according to tumor progression.

Local medication treatment is a new method and provides new hope for the treatment of malignant glioma. Real-time MRI tracer technology will help study the biological basis of glioma cell interstitial diffusion, which can contribute to further development of local treatment of malignant gliomas.

In conclusion, ECS parameters were altered with C6 glioma progression from increased ECM content. These findings provide a basis for understanding the glioma microenvironment, which can help improve interstitial drug delivery to the brain for the treatment of gliomas.
